# Efficacy and safety of traction-assisted endoscopic submucosal dissection for superficial gastric neoplasms: a meta-analysis of randomized controlled trials

**DOI:** 10.3389/fonc.2026.1824135

**Published:** 2026-05-29

**Authors:** Zhen-guang Zhao, Wen-guang Xu, Rui Zhang, Xiao-mei Li, Chen Yang, Li Wang, Jing Xie

**Affiliations:** 1 Department of Gastroenterology, Beijing Puxiang Hospital of Traditional Chinese Medicine (TCM), Beijing, China; 2 Department of Gastroenterology, The Affiliated Qingdao Third People’s Hospital of Qingdao University, Qingdao, Shandong, China; 3 Department of Emergency Medicine, Rocket Force Characteristic Medical Center, Beijing, China; 4 Laoshan Medical District of No. 971 Hospital of Chinese Navy, Qingdao, Shandong, China; 5 Department of Oncology, Sunshine Union Hospital, Weifang, China; 6 Department of Gastroenterology, Rocket Force Characteristic Medical Center, Beijing, China

**Keywords:** endoscopic submucosal dissection, gastric neoplasm, meta-analysis, randomized controlled trials, traction-assisted

## Abstract

**Background:**

Endoscopic submucosal dissection (ESD) remains technically demanding for superficial gastric neoplasms, particularly during submucosal layer dissection which is often time-consuming and associated with procedural risks. While traction-assisted ESD has been proposed to enhance visualization and facilitate safer dissection, current evidence regarding its efficacy and safety remains inconsistent.

**Methods:**

We conducted a comprehensive meta-analysis of randomized controlled trials (RCTs) comparing traction-assisted versus conventional ESD for superficial gastric neoplasms. Primary outcome was procedure time, with secondary outcomes were resection completeness (*en bloc* and R0 rates). Safety outcomes focused on adverse events (perforation and delayed bleeding). Subgroup analyses were performed by lesion size, lesion location, operator experience level and study design.

**Results:**

Seven RCTs involving 1476 patients were included. Meta-analysis demonstrated that traction-assisted ESD significantly reduced procedure time compared to conventional ESD (SMD: -0.23, 95% CI: -0.44 to -0.02; *P* = 0.034), with particularly pronounced benefits for lesions ≤20mm (SMD: -0.39, 95% CI: -0.69 to -0.09, *P* = 0.011) and those located at the greater curvature of the upper/middle stomach (SMD: -0.58, 95% CI: -0.98 to -0.19; *P* = 0.004). However, the benefit was operator-dependent: traction-assisted ESD significantly reduced procedure time in beginners (SMD: -0.22, 95% CI: -0.40 to -0.04; *P* = 0.019) but not in experts (SMD: -0.08, 95% CI: -0.22 to 0.06; *P* = 0.250). Resection quality remained comparable between groups, with similar *en bloc* (RR: 1.00, 95% CI: 0.99-1.01; *P* = 1.00) and R0 resection rates (RR: 1.01, 95% CI: 0.99-1.03; *P* = 0.156). Importantly, traction-assisted ESD showed a 73% reduction in perforation risk (RR: 0.27, 95% CI: 0.08-0.91; *P* = 0.034) while maintaining equivalent delayed bleeding rate (RR: 0.97, 95% CI: 0.59-1.60; *P* = 0.920).

**Conclusions:**

Traction-assisted ESD appears to be an efficient and safe technique for superficial gastric neoplasms, particularly for small lesions (≤20 mm) and those in technically challenging locations. Notably, the procedure time benefit was significant only among less experienced operators, suggesting that traction may be most valuable for lowering the technical threshold in non-expert hands. However, given the observed sensitivity to individual studies, current evidence should be interpreted with caution. Further large-scale, multi-center RCTs are warranted to confirm these findings.

## 1 Introduction

Endoscopic submucosal dissection (ESD) has emerged as a cornerstone in the minimally invasive treatment of superficial gastric neoplasms, offering a curative option for early-stage lesions (1–3). This technique allows for the *en bloc* resection of large or irregularly shaped tumors, providing superior pathological assessment and reducing local recurrence rates compared to endoscopic mucosal resection (4, 5). However, despite its clinical advantages, ESD remains technically demanding, particularly during the dissection of the submucosal layer. Challenges such as inadequate visualization, limited tissue tension, and the complex anatomy of the stomach often prolong procedure times and increase the risk of complications, including perforation and delayed bleeding (6–8). These factors have hindered the widespread adoption of ESD, especially in regions with less endoscopic expertise.

To address these limitations, traction-assisted ESD techniques have been developed, aiming to improve submucosal visualization and facilitate safer, more efficient dissections. Various traction methods, such as clip-line, clip-snare, grasping forceps, and spring-and-loop with clip systems, have been introduced to provide consistent tissue tension and enhance endoscopic maneuverability (9–12). While preliminary studies suggest that these techniques may shorten procedure times and reduce adverse events, the evidence remains inconsistent. Some randomized controlled trials (RCTs) report significant benefits (13, 14), whereas others show no clear advantage (15, 16), possibly due to variations in lesion characteristics, traction methods, or operator experience. Additionally, many existing studies are limited by small sample sizes, underscoring the need for a comprehensive evaluation of traction-assisted ESD’s efficacy and safety in superficial gastric neoplasms.

Thus, we conducted this meta-analysis of RCTs to synthesize the available evidence, providing a robust assessment of whether traction-assisted ESD outperforms conventional ESD in terms of procedure time, resection completeness (*en bloc* and R0 rates), and complication rates (perforation and delayed bleeding) in superficial gastric neoplasms. By stratifying outcomes based on lesion size and location, we aim to identify specific scenarios where traction-assisted ESD offers the greatest clinical benefit.

## 2 Materials and methods

### 2.1 Search strategy

A systematic literature search was conducted across multiple electronic databases, including PubMed, Embase, and the Cochrane Central Register of Controlled Trials (CENTRAL), to identify relevant studies published up to May 2025. The search strategy employed a combination of Medical Subject Headings (MeSH) terms and free-text keywords related to endoscopic submucosal dissection, traction techniques, and gastric neoplasms (**Supplementary Tables 1–3**). RCTs comparing traction-assisted ESD with conventional ESD in superficial gastric neoplasms were identified. This meta-analysis was conducted in accordance with the Preferred Reporting Items for Systematic Reviews and Meta-Analyses (PRISMA) guidelines (17).

### 2.2 Selection criteria

Two investigators independently evaluated all candidate studies for eligibility. The primary outcome in this meta-analysis was procedure time, with secondary efficacy outcomes including *en bloc* resection rate and R0 resection rate. Procedure time was calculated from initial submucosal injection to complete lesion removal. *En bloc* resection referred to complete tumor removal in one intact specimen. R0 resection was defined as *en bloc* excision with histologically confirmed negative lateral and deep margins. Safety assessments focused on two key complications: intraprocedural perforation and postprocedural delayed bleeding. Eligible studies were restricted to RCTs that directly compared traction-assisted ESD with conventional ESD in patients with superficial gastric neoplasms and reported at least one of our prespecified outcomes. Conference abstracts and non-peer-reviewed materials were systematically excluded. All discrepancies were resolved by discussion.

### 2.3 Data extraction

Two investigators independently extracted data using a standardized form, systematically collecting key information from each eligible study including study characteristics (first author, publication year, study design [single-center/multicenter], and country of origin), patient and lesion parameters (sample size, lesion diameter, and lesion location), technical details (specific traction methods such as clip-line or clip-snare), and outcome measures (procedure time, *en bloc* resection rate, R0 resection rate, perforation incidence, and delayed bleeding frequency) to ensure consistency in data collection. For studies reporting continuous variables as median (with range or interquartile range), these values were transformed to mean ± standard deviation using established statistical methods (18) to facilitate meta-analysis. Any discrepancies in data extraction were resolved through discussion. The corresponding authors of included studies were contacted if necessary.

### 2.4 Methodological quality evaluation

To assess the methodological rigor of the included studies, a comprehensive bias evaluation was conducted following the Cochrane Collaboration Reviews’ Handbook (19). Two independent investigators systematically appraised each study across seven critical domains: randomization process, allocation concealment, blinding of participants and personnel, blinding of outcome assessment, completeness of outcome data, selective reporting, and other potential sources of bias. For each domain, investigators assigned one of three judgments: “low risk,” “high risk,” or “unclear,” with any discrepancies resolved through discussion.

### 2.5 Statistical analyses

Statistical analyses were performed using Stata 12.0 (Stata Corporation, College Station, TX, USA). For binary outcome measures, we computed pooled risk ratios (RR) with corresponding 95% confidence intervals (CI), while continuous variables were analyzed using standardized mean differences (SMD) with 95% CI. The degree of between-study heterogeneity was assessed through *I²* statistics, with values exceeding 50% (coupled with a significance threshold of *P* < 0.10) considered indicative of meaningful heterogeneity (20). A leave-one-out sensitivity analysis was performed to assess the influence of individual studies on the pooled estimate. To reveal the potential source of statistical heterogeneity, we performed subgroup meta-analysis based on lesion size (≤20mm versus >20mm), lesion location (upper, middle, and lower thirds of the stomach) (21), operator experience level (beginner vs. expert), and study design (single-center vs. multi-center). Regardless of heterogeneity levels, random-effects models was used for all meta-analyses to ensure conservative estimates (22). Statistical significance was defined as *P* < 0.05.

## 3 Results

### 3.1 Study selection

**Figure 1:**
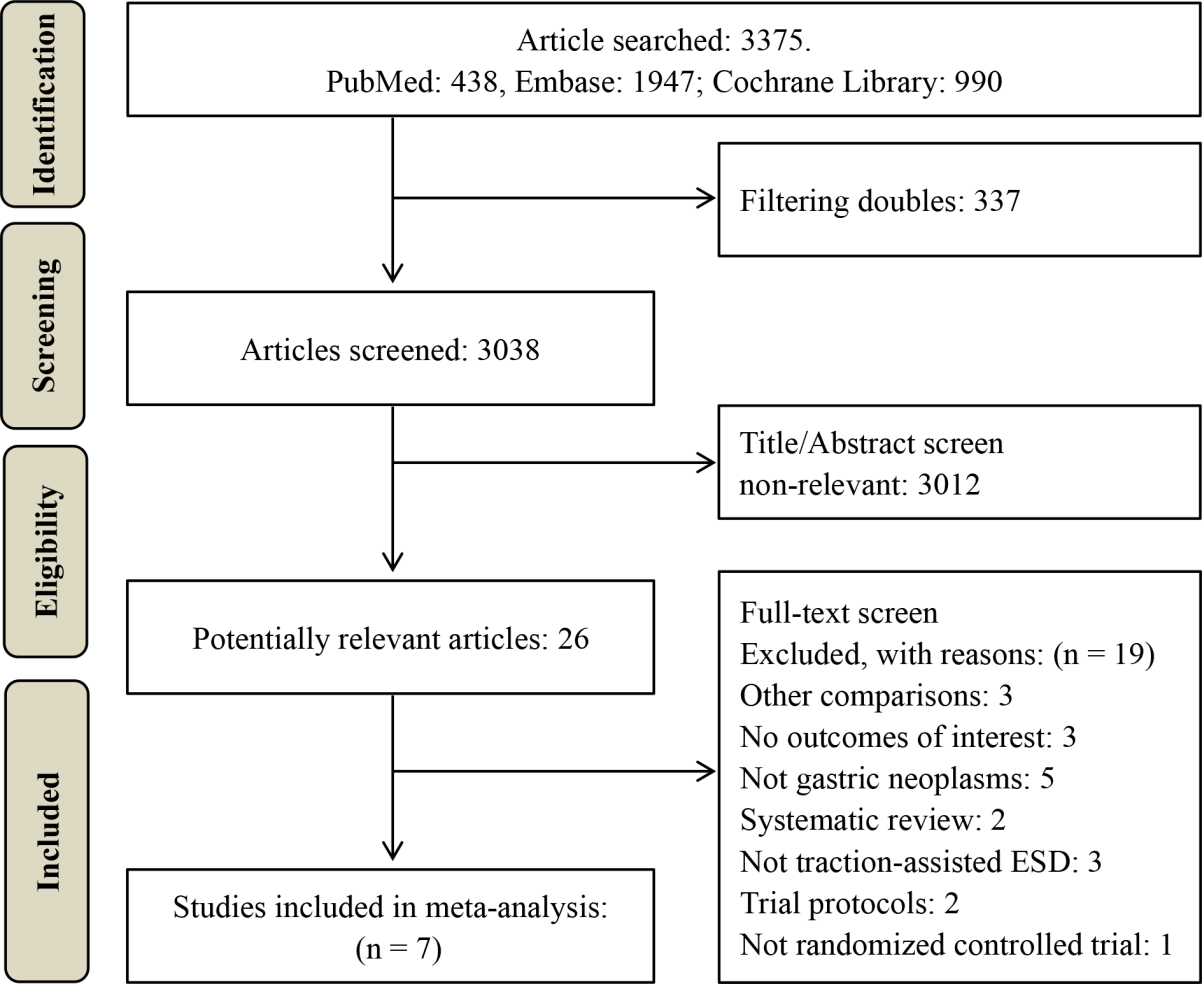
Study selection flow diagram.

Our systematic search initially identified 3375 potentially eligible articles from electronic databases. Following removal of 337 duplicate records, 3038 studies proceeded to initial screening. After evaluation of titles and abstracts, we excluded 3012 publications that failed to meet inclusion criteria. The remaining 26 articles underwent comprehensive full-text assessment, leading to the exclusion of an additional 19 studies due to various reasons. Ultimately, seven RCTs comprising 1476 superficial gastric neoplasm patients (733 in traction-assisted ESD group and 743 in conventional ESD group) were included in this meta-analysis (13–16, 23–25), as illustrated in **Figure 1**.

### 3.2 Characteristics of the included studies

The characteristics of the seven included RCTs are systematically presented in **Table 1**. These studies exhibited considerable variation in sample size, ranging from 51 to 635 participants. Geographically, the majority of trials (n=5) were conducted in Japan (14–16, 23, 24), with single contributions from China (13) and Korea (25). Regarding study design, five trials employed a single-center approach (13, 14, 16, 24, 25), while two larger-scale investigations were conducted across multiple centers (15, 23).

**Table 1:** Summary characteristics of the included randomized controlled trials.

Author	Publication year	Enrolled period	Sample size (traction/control)	Lesion diameter (traction/control, mm)	Traction method	Operator experience	Study design	Country
Bi et al. (13)	2024	2022-2024	40/40	15.0 (10.3, 17.8); 15.0 (13.0, 20.0)	Per-nasal clip-and-snare	Single expert endoscopist	Single-center RCT	China
Kinoshita et al. (16)	2024	2021-2023	73/75	14.9 ± 9.7; 14.8 ± 11.0	Clip-and-thread	Mixed (beginner/expert)	Single-center RCT	Japan
Hasatani et al. (23)	2022	2017-2020	186/192	13.2 ± 8.8; 13.8 ± 9.5	Clip-and-snare	Mixed (beginner/expert)	Multicenter RCT	Japan
Nagata et al. (14)	2021	2018-2019	40/40	15.9 ± 10.3; 15.7 ± 10.4	Spring-and-loop with clip	Single expert endoscopist	Single-center RCT	Japan
Yoshida et al. (15)	2018	2015-2016	319/316	15.7 ± 10.1; 15.5 ± 8.9	Dental floss clip	Mixed (beginner/expert)	Multicenter RCT	Japan
Ban et al. (24)	2018	2015-2016	49/55	19.7 ± 15.1; 17.5 ± 11.8	Clip-flap	Mixed (beginner/expert)	Single-center RCT	Japan
Ahn et al. (25)	2013	2010-2011	26/25	20.5 ± 7.9; 19.4 ± 6.5	Trans-nasal endoscope	Beginner only	Single-center RCT	Korea

RCT, randomized controlled trial.

### 3.3 Methodological quality evaluation

All seven RCTs (13–16, 23–25) provided explicit descriptions of their randomization procedures, with four studies (13–15, 23) additionally detailing allocation concealment methods. Due to the inherent nature of endoscopic interventions, none of the trials implemented blinding for endoscopists, while only one study (13) reported participant blinding. All included studies demonstrated low risk of bias across critical domains, including completeness of outcome data, absence of selective reporting, and control for other potential biases (**Supplementary Figure 1**).

### 3.4 Procedure time and subgroup meta-analysis

**Figure 2:**
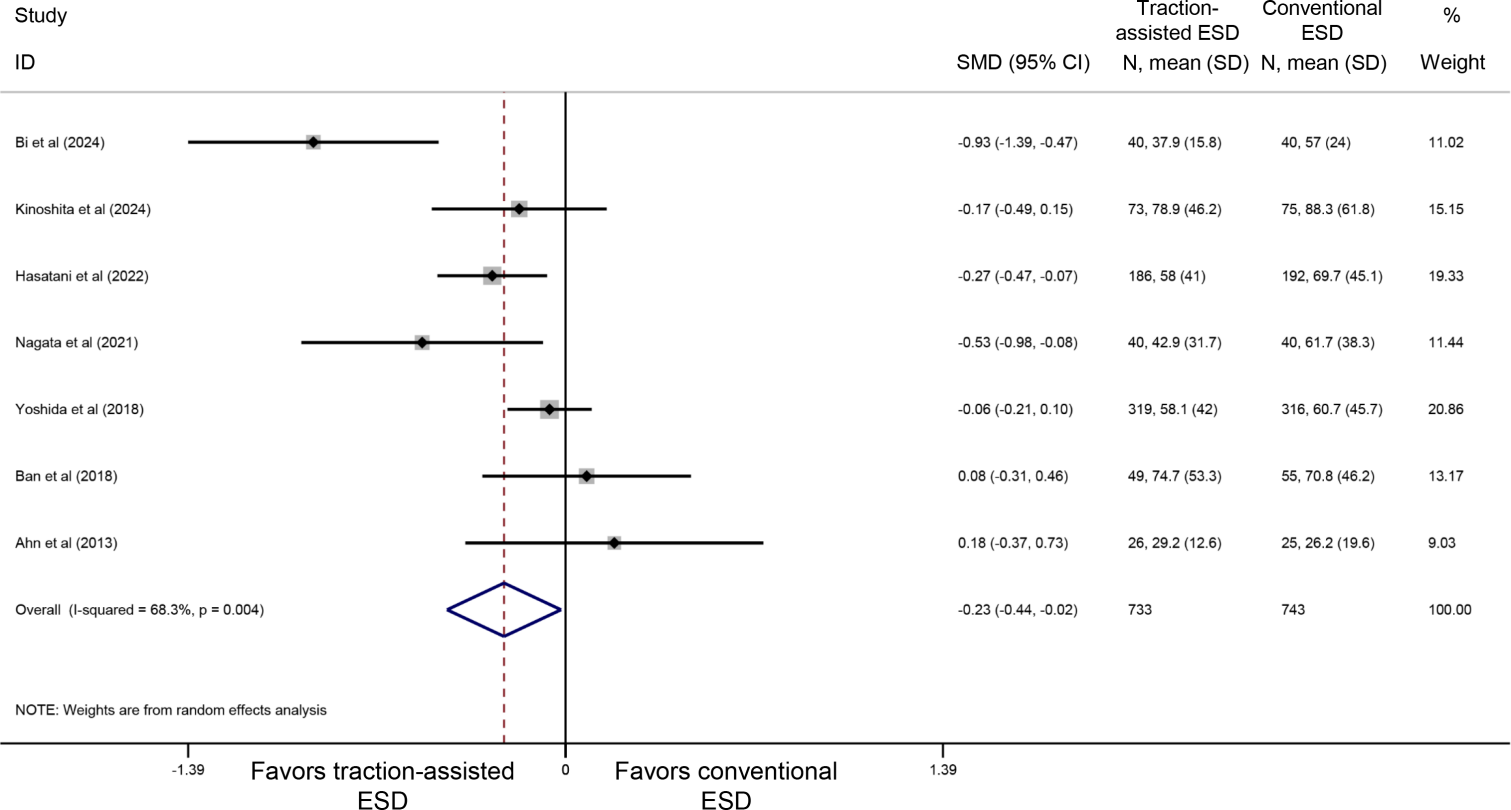
Forest plot demonstrating the comparative analysis of procedure time between traction-assisted and conventional ESD for superficial gastric neoplasms. For each study, descriptive statistics (N, mean, standard deviation [SD]) are shown for both groups, along with the standardized mean difference (SMD) and 95% confidence intervals (CI) for each study and the pooled estimate. ESD: endoscopic submucosal dissection.

The pooled analysis of all seven included studies (13–16, 23–25) demonstrated that traction-assisted ESD significantly reduced procedure time compared to conventional ESD (SMD: −0.23, 95% CI: −0.44 to −0.02; *P* = 0.034; **Figure 2**). While the overall pooled estimate favored traction-assisted ESD, the sensitivity analysis revealed that the statistical significance was not robust to the exclusion of certain studies. Specifically, after omitting the studies by Bi et al. (2024), Kinoshita et al. (2024), Hasatani et al. (2022), or Nagata et al. (2021), the pooled SMD shifted from statistically significant to non-significant (**Supplementary Table 4**).

**Figure 3:**
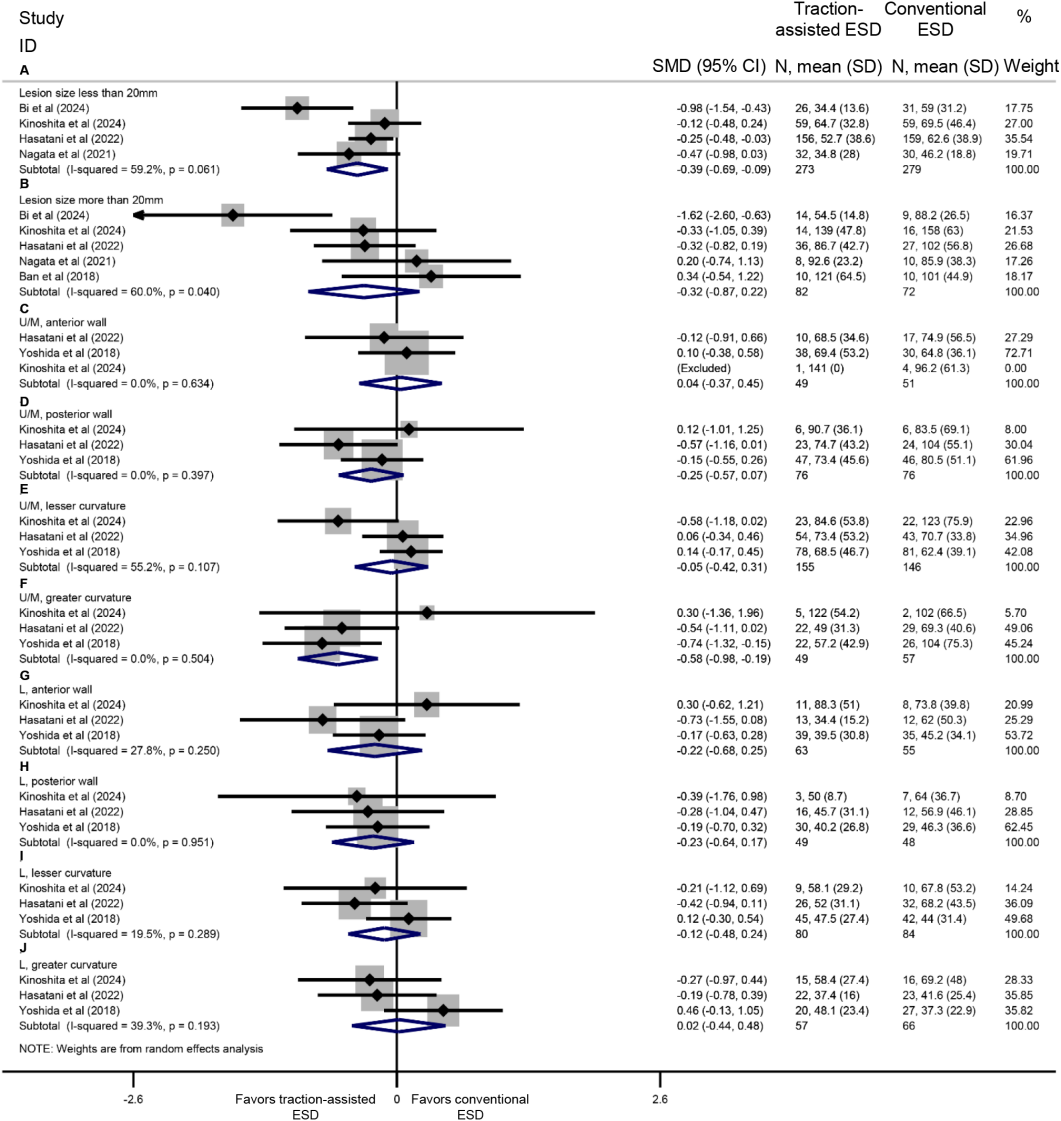
Subgroup analysis of procedure time differences stratified by **(A)** lesions ≤20mm versus **(B)** lesions >20mm in diameter, and by anatomical location: **(C)** upper/middle (U/M) anterior wall, **(D)** U/M posterior wall, **(E)** U/M lesser curvature, **(F)** U/M greater curvature, **(G)** lower **(L)** anterior wall, **(H)** L posterior wall, **(I)** L lesser curvature, and **(J)** L greater curvature. All analyses compare traction-assisted versus conventional ESD. ESD, endoscopic submucosal dissection.

Subgroup analyses revealed important variations based on lesion characteristics. For smaller lesions (≤20 mm), traction assistance provided a more pronounced reduction in procedure time (SMD: −0.39, 95% CI: −0.69 to −0.09; *P* = 0.011), whereas no significant benefit was observed for larger lesions (>20 mm; SMD: −0.32, 95% CI: −0.87 to 0.22; *P* = 0.247). Anatomically, the greatest advantage was seen in lesions located at the greater curvature of the upper/middle stomach (SMD: −0.58, 95% CI: −0.98 to −0.19; *P* = 0.004), while no significant differences were detected for other locations, including the anterior/posterior walls and lesser curvature of the upper/middle stomach, or any examined sites in the lower stomach (**Figure 3**).

Subgroup analyses based on operator experience level revealed that traction-assisted ESD significantly reduced procedure time in beginners (SMD: −0.22, 95% CI: −0.40 to −0.04; *P* = 0.019) but not in expert endoscopists (SMD: −0.08, 95% CI: −0.22 to 0.06; *P* = 0.250) (**Supplementary Figure 2**). In contrast, subgroup analyses based on study design showed no significant differences between the traction-assisted ESD and conventional ESD groups in either single−center or multi−center RCTs (single−center: SMD: −0.28, 95% CI: −0.64 to 0.09, *P* = 0.144; multi−center: SMD: −0.15, 95% CI: −0.36 to 0.05, *P* = 0.142) (**Supplementary Figure 3**).

### 3.5 *En bloc* resection rate and R0 resection rate

**Figure 4:**
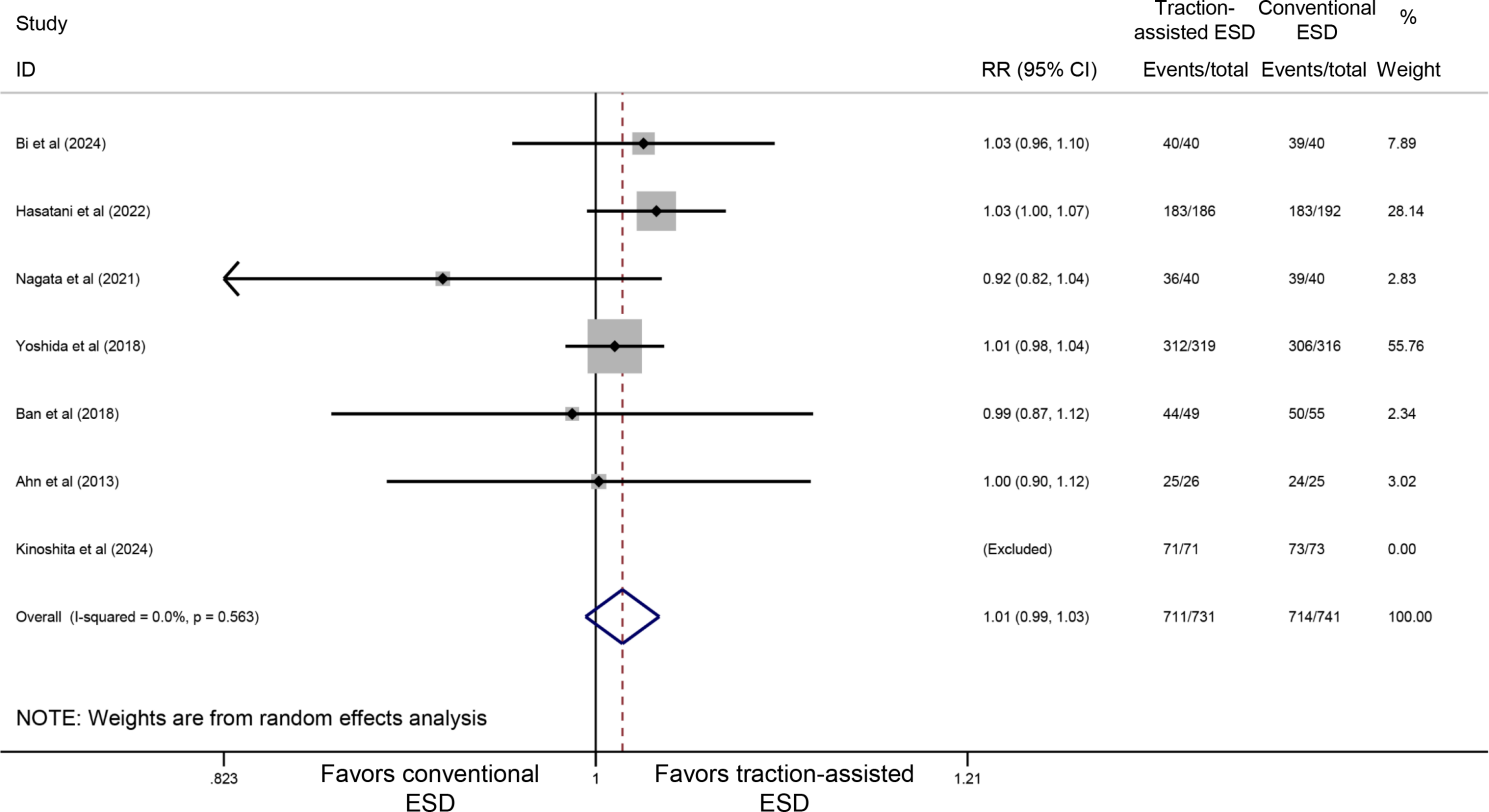
Forest plot of R0 resection rates between traction-assisted and conventional ESD. For each study, the number of events and total sample size are shown for both groups, along with the risk ratio (RR) and 95% confidence interval (CI) for each individual study and the pooled estimate. ESD, endoscopic submucosal dissection.

All seven included studies (13–16, 23–25) provided complete data on both *en bloc* and R0 resection rates. Meta-analysis revealed comparable efficacy between traction-assisted and conventional ESD techniques, with no statistically significant differences observed in either *en bloc* resection rates (RR: 1.00, 95% CI: 0.99 to 1.01; *P* = 1.00; **Supplementary Figure 4**) or R0 resection rates (RR: 1.01, 95% CI: 0.99 to 1.03; *P* = 0.156; **Figure 4**).

### 3.6 Perforation and delayed bleeding

**Figure 5:**
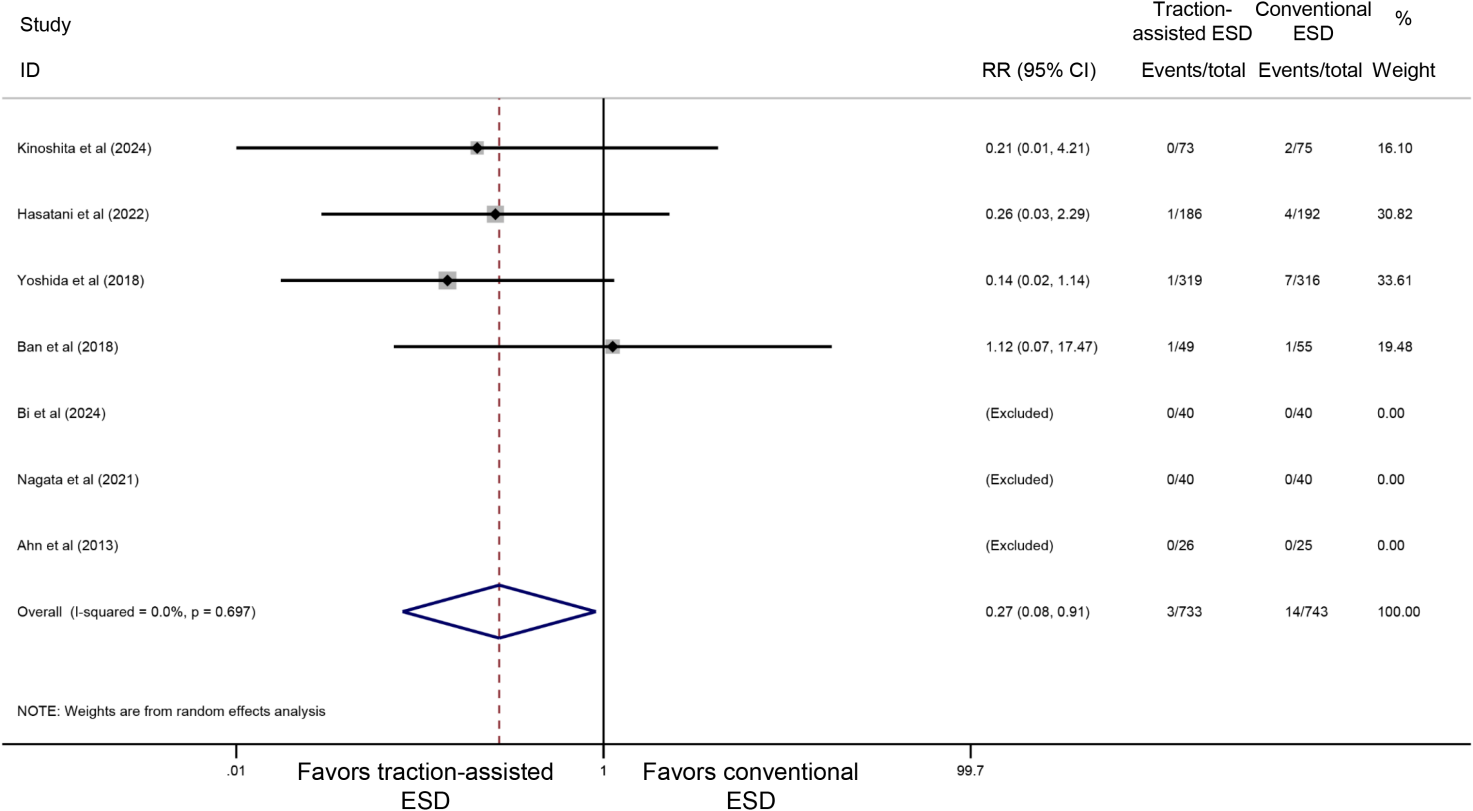
Forest plot of perforation rates comparing traction-assisted ESD with conventional ESD. For each study, events and total sample size are presented for both groups, along with the risk ratio (RR) and 95% confidence interval (CI) for each study and the pooled estimate. ESD, endoscopic submucosal dissection.

**Figure 6:**
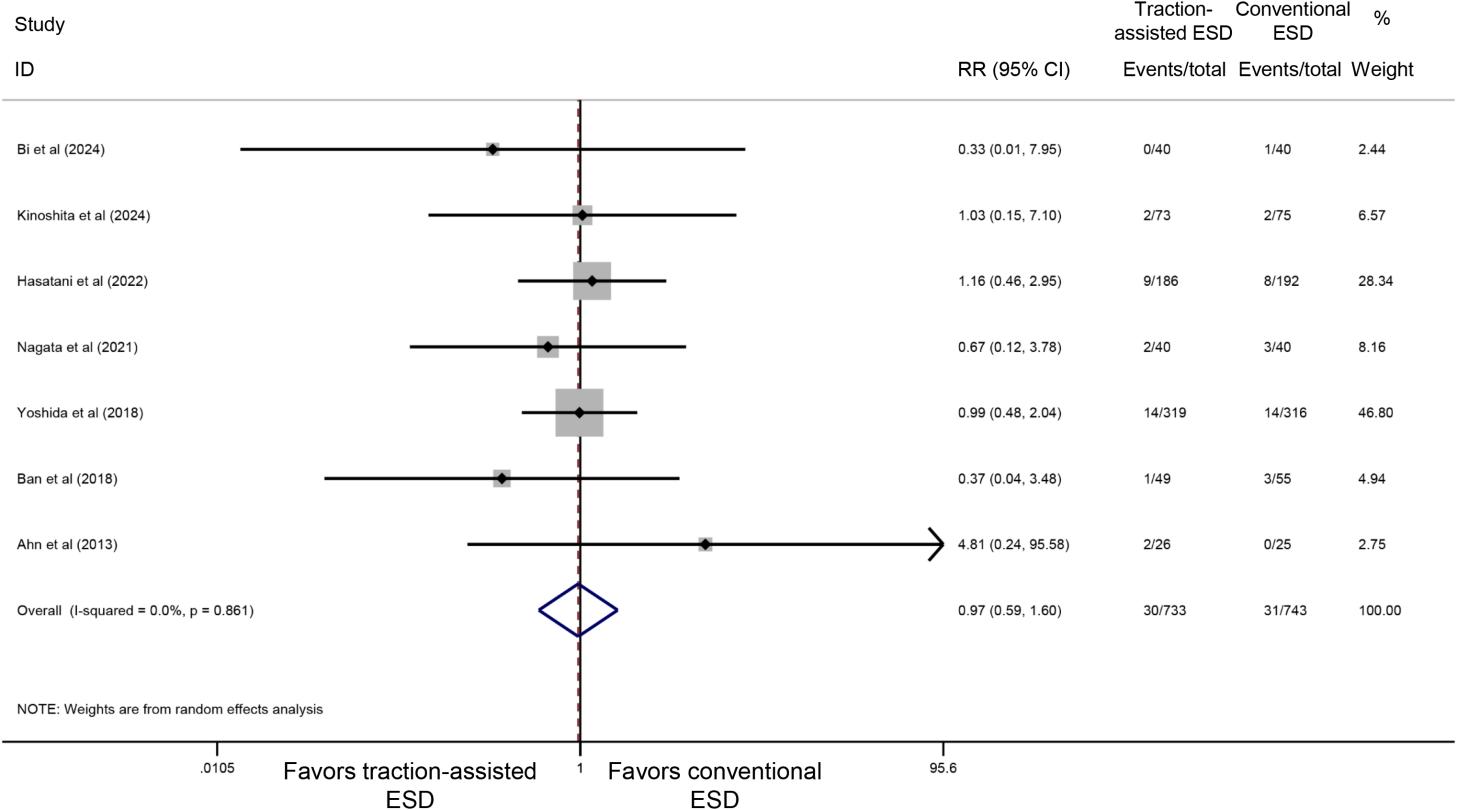
Forest plot of delayed bleeding rates comparing traction-assisted ESD with conventional ESD. For each study, events and total sample size are presented for both groups, along with the risk ratio (RR) and 95% confidence interval (CI) for each study and the pooled estimate. ESD, endoscopic submucosal dissection.

Safety outcomes were systematically evaluated across all seven included studies (13–16, 23–25), with meta-analysis demonstrating a significant reduction in perforation risk with traction-assisted ESD compared to conventional ESD (RR: 0.27, 95% CI: 0.08 to 0.91; *P* = 0.034; **Figure 5**). The pooled perforation rate was 0.4% (3/733) in the traction-assisted ESD group versus 1.9% (14/743) in the conventional ESD group. This corresponds to an absolute risk reduction of 1.5% and a number needed to treat (NNT) of 67 to prevent one perforation. This three-fold decrease in perforation incidence highlights a key safety advantage of the traction-assisted approach. In contrast, rate of delayed bleeding showed no statistically significant difference between the two methods (RR: 0.97, 95% CI: 0.59 to 1.60; *P* = 0.920; **Figure 6**).

## 4 Discussion

ESD remains the gold-standard treatment for superficial gastric neoplasms, offering superior *en bloc* resection rates and precise histological evaluation. However, its widespread adoption has been limited by technical complexity and prolonged procedure times (26, 27). Our meta-analysis demonstrates that traction-assisted ESD significantly reduces procedure duration while maintaining comparable *en bloc* and R0 resection rates to conventional ESD, with the added benefit of a 73% lower perforation risk. These findings suggest that traction assistance enhances both efficiency and safety without compromising therapeutic quality.

The upper/middle stomach presents unique anatomical challenges for ESD (28), including confined working space near the esophagogastric junction, thicker mucosal layers leading to suboptimal submucosal lifting, and tangential approach angles that compromise visualization and instrument maneuverability (29, 30). Our subgroup analysis revealed that traction-assisted ESD was particularly advantageous for lesions in the greater curvature of the upper/middle stomach, where conventional ESD is most technically demanding. This anatomical specificity highlights the clinical value of traction techniques in overcoming the inherent difficulties of these challenging locations, potentially expanding the feasibility of endoscopic resection for proximal gastric lesions that would otherwise require alternative approaches.

Our meta-analysis revealed a significant reduction in procedure time with traction-assisted ESD for lesions ≤20 mm, but not for larger lesions >20 mm. This size-dependent efficacy can be attributed to following technical factors. The gradual decrease in traction force during submucosal dissection - resulting from progressive reduction in the distance between the traction and anchor sites - appears to be adequately maintained throughout resection of smaller lesions, whereas the sustained tension required for larger lesions diminishes before complete resection is achieved. This biomechanical limitation suggests that current traction methods may be inherently better suited for smaller lesions, where the procedure duration aligns with the effective maintenance of traction force.

To optimize traction-assisted ESD for larger lesions, procedural modifications such as dynamic repositioning of traction points or simultaneous multi-point fixation could theoretically maintain adequate tension throughout extended dissections. However, the current evidence remains limited by the relatively small proportion of large lesions included in our analysis. This underscores the need for future prospective studies specifically evaluating traction techniques in larger gastric neoplasms, with particular attention to innovative approaches for sustained traction maintenance during prolonged procedures (31).

Subgroup analyses revealed that the benefit of traction-assisted ESD on procedure time varied substantially according to operator experience level. In studies involving beginner operators, traction-ESD significantly reduced procedure time, whereas no significant benefit was observed among expert operators. This pattern suggests that the traction technique may be most valuable for lowering the technical threshold for less experienced endoscopists, rather than further enhancing the performance of experts. This finding represents a plausible “ceiling effect,” given that expert operators have already optimized their conventional dissection skills.

Our subgroup analysis based on study design (single−center vs. multi−center) did not reveal statistically significant differences, although point estimates favored traction−assisted ESD in both settings. We propose that operator experience level may serve as the more clinically relevant effect modifier. The rationale is as follows. First, the modest benefits observed in multi−center RCTs may be attributable to the predominance of expert endoscopists in these trials (74.5% in Yoshida’s study and 58.7% in Hasatani’s study). Second, among the single−center studies, two trials (Bi et al., 2024 and Nagata et al., 2021) exclusively enrolled expert operators. This may explain the observed trend toward a difference that did not reach statistical significance for traction−assisted ESD (SMD: −0.28, 95% CI: −0.64 to 0.09).

Maintaining optimal submucosal visualization is crucial for minimizing complications during ESD procedures. Our analysis demonstrates that traction-assisted ESD offers a significant reduction in perforation risk compared to conventional techniques. However, its clinical significance must be interpreted in the context of the low baseline event rate. The absolute risk reduction of 1.5% means that approximately 67 patients would need to be treated with traction−assisted ESD to prevent one perforation. This absolute benefit is clinically meaningful for several reasons. First, gastric ESD perforation is a serious adverse event associated with prolonged hospitalization, need for endoscopic or surgical closure, intravenous antibiotics, and fasting, leading to substantial patient morbidity and healthcare costs. Second, the baseline perforation risk varies considerably across clinical settings. In high−risk subgroups, such as lesions located in the upper stomach (32, 33), larger lesions (>20 mm), or procedures performed by less experienced operators, the baseline risk may be substantially higher. In these scenarios, the absolute risk reduction would be proportionally greater, resulting in a lower NNT. Third, for high−volume endoscopists or institutions performing hundreds of gastric ESDs annually, a 1.5% absolute risk reduction translates into several prevented serious complications each year, with meaningful cumulative benefits.

Although the overall effect size for procedure time reduction was modest (SMD: -0.23), this translates to an absolute time saving of approximately 10–15 minutes per procedure. From a clinical perspective, this reduction may contribute to decreased sedation-related risks, reduced operator fatigue, and improved endoscopic suite efficiency. More importantly, traction-ESD demonstrated a clinically substantial 73% reduction in perforation risk, which represents a more critical patient safety outcome. Moreover, subgroup analyses revealed considerably larger effect sizes in clinically relevant scenarios, including lesions ≤20 mm and those located at the greater curvature of the upper/middle stomach. Taken together, while the overall time reduction is modest, the technique offers clinically valuable benefits, particularly in terms of safety and for specific high-need patient subgroups.

Notably, comparable rates of delayed bleeding between traction-assisted and conventional ESD indicate that traction application preserves hemostatic integrity at the resection site. This safety profile confirms that while traction enhances procedural control, it does not introduce new risks or compromise vascular sealing. Together, these findings establish traction-assisted ESD as an advanced technique that reduces major complications without creating additional adverse effects.

Several limitations of this meta-analysis should be considered. First, the inclusion of only seven RCTs, though rigorously selected, may limit the generalizability of our findings. A notable methodological challenge was the inherent impossibility of blinding endoscopists to the intervention, potentially introducing performance bias during procedure execution and outcome assessment. Second, substantial clinical heterogeneity existed across studies regarding two key dimensions: (1) lesion characteristics, including variations in size distribution and anatomical locations, and (2) technical approaches, encompassing diverse traction devices (clip-line, clip-snare, etc.), application methods (oral vs nasal routes), and traction vectors (varying directions and forces). These technical variations, while reflecting real-world practice, may account for some outcome discrepancies. Consistently, sensitivity analysis indicated that the statistical significance of the pooled effect was not stable. This suggests that our findings may be driven by a subset of studies. The potential instability may be attributed to heterogeneity in patient selection criteria, lesion location, operator experience, or specific traction techniques employed across studies. Therefore, the current evidence should be interpreted with caution. Furthermore, our analysis was limited to direct comparisons between traction-assisted and conventional ESD; the relative efficacy among different traction modalities remains an important area for future comparative studies.

In summary, our meta-analysis suggests that traction-assisted ESD may offer certain advantages over conventional ESD for superficial gastric neoplasms, including a modest reduction in procedure time and a lower risk of perforation. The potential benefits appear more promising in specific subgroups, such as smaller lesions (≤20 mm) and anatomically challenging locations (e.g., the greater curvature of the upper/middle stomach), although these subgroup findings should be interpreted cautiously given their exploratory nature. Notably, the procedure time benefit was significant only among beginner operators but not among experts, suggesting that traction-ESD may be most valuable for less experienced endoscopists. Given the modest effect sizes and the sensitivity of the overall findings to individual studies, further large-scale, multi-center RCTs are warranted to confirm these observations.

## Data Availability

The original contributions presented in the study are included in the article/**Supplementary Material**. Further inquiries can be directed to the corresponding authors.
