# Supplementary material for: Efficacy and safety of traction-assisted endoscopic submucosal dissection for superficial gastric neoplasms: a meta-analysis of randomized controlled trials
Source: Front Oncol. 2026 May 29;16:1824135. doi: 10.3389/fonc.2026.1824135 (PMC13259855; doi:10.3389/fonc.2026.1824135)
Supplement: Supplementary file 1 [file DataSheet1.pdf]

Table S1 Search criterion of PubMed (from inception to May 15, 2025)

No.	Query Results	Results
#36	#8 and #32 and #35	438
#35	#33 or #34	1887172
#34	Search: "Randomized Controlled Trial" [Publication Type] OR "Randomized Controlled Trials as Topic"[Mesh] Sort by: Most Recent	818808
#33	Search: random*[Text Word]	1885385
#32	#9 or #10 or #11 or #12 or #13 or #14 or #15 or #16 or #17 or #18 or #19 or #20 or #21 or #22 or #23 or #24 or #25 or #26 or #27 or #28 or #29 or #30 or #31	2881806
#31	Search: ring thread*[Title/Abstract]	25
#30	Search: band*[Title/Abstract]	381778
#29	Search: sinker*[Title/Abstract]	151
#28	Search: anchor*[Title/Abstract]	109267
#27	Search: magnetic*[Title/Abstract]	682375
#26	Search: transnasal endoscope*[Title/Abstract]	66
#25	Search: double scope*[Title/Abstract]	34
#24	Search: preloop*[Title/Abstract]	35
#23	Search: snare*[Title/Abstract]	11204
#22	Search: string*[Title/Abstract]	63469
#21	Search: thread*[Title/Abstract]	23633
#20	Search: grasping forcep*[Title/Abstract]	482
#19	Search: forcep*[Title/Abstract]	15925
#18	Search: dental floss[Title/Abstract]	825
#17	Search: endoclip*[Title/Abstract]	557
#16	Search: hemoclip*[Title/Abstract]	632
#15	Search: clip*[Title/Abstract]	39292
#14	Search: tow[Title/Abstract]	892
#13	Search: guide*[Title/Abstract]	1084737

#12	Search: haul*[Title/Abstract]	2603
#11	Search: drag*[Title/Abstract]	17443
#10	Search: pull*[Title/Abstract]	68259
#9	Search: tract*[Title/Abstract]	514598
#8	#1 or #2 or #3 or #4 or #5 or #6 or #7	23007
#7	Search: "Endoscopic Mucosal Resection"[Mesh] Sort by: Most Recent	4739
#6	Search: EMR[Title/Abstract]	10752
#5	Search: endoscopic mucosal resection[Title/Abstract]	4076
#4	Search: ESD[Title/Abstract]	7183
#3	Search: endoscopic dissection[Title/Abstract]	261
#2	Search: submucosal dissection[Title/Abstract]	8136
#1	Search: endoscopic submucosal dissection[Title/Abstract]	7913

Table S2 Search criterion of Embase (from inception to May 15, 2025)

No.	Query Results	Results
#36	#8 AND #32 AND #35	1947
#35	#33 OR #34	2698574
#34	'randomized controlled trial'/exp OR 'randomized controlled trial'	1376323
#33	random*:ab,ti,kw	2430233
#32	#9 OR #10 OR #11 OR #12 OR #13 OR #14 OR #15 OR #16 OR #17 OR #18 OR #19 OR #20 OR #21 OR #22 OR #23 OR #24 OR #25 OR #26 OR #27 OR #28 OR #29 OR #30 OR #31	4937478
#31	ring AND thread*:ab,ti	882
#30	band*:ab,ti	403110
#29	sinker*:ab,ti	151
#28	anchor*:ab,ti	127548
#27	magnetic*:ab,ti	772577
#26	transnasal AND endoscope*:ab,ti	783
#25	double AND scope*:ab,ti	3159
#24	preloop*:ab,ti	43
#23	snare*:ab,ti	17295
#22	string*:ab,ti	78739
#21	thread*:ab,ti	27886
#20	grasping AND forcep*:ab,ti	1455
#19	forcep*:ab,ti	26132
#18	dental AND floss*:ab,ti	2154
#17	endoclip*:ab,ti	1466
#16	hemoclip*:ab,ti	1631
#15	clip*:ab,ti	59644
#14	tow*:ab,ti	1343721
#13	guide*:ab,ti	1607276
#12	haul*:ab,ti	2927

#11	drag*:ab,ti	22130
#10	pull*:ab,ti	87003
#9	tract*:ab,ti	698448
#8	#1 OR #2 OR #3 OR #4 OR #5 OR #6 OR #7	65038
#7	'endoscopic submucosal dissection'/exp OR 'endoscopic submucosal dissection'	17766
#6	emr:ab,ti	27333
#5	endoscopic AND mucosal AND resection:ab,ti	14242
#4	esd:ab,ti	14923
#3	endoscopic AND dissection:ab,ti	27181
#2	submucosal AND dissection:ab,ti	16357
#1	endoscopic AND submucosal AND dissection:ab,ti	15641

Table S3 Search criterion of Cochrane Library (from inception to May 15, 2025)

No.	Query Results	Results
#1	(endoscopic submucosal dissection):ti,ab,kw	1206
#2	(submucosal dissection):ti,ab,kw	1246
#3	(endoscopic dissection):ti,ab,kw	1665
#4	(ESD):ti,ab,kw	1316
#5	(endoscopic mucosal resection):ti,ab,kw	933
#6	(EMR):ti,ab,kw	1571
#7	MeSH descriptor: [Endoscopic Mucosal Resection] explode all trees	236
#8	#1 or #2 or #3 or #4 or #5 or #6 or #7	3701
#9	(tract*):ti,ab,kw	54116
#10	(pull*):ti,ab,kw	4043
#11	(drag*):ti,ab,kw	1033
#12	(haul*):ti,ab,kw	92
#13	(guide*):ti,ab,kw	122969
#14	(tow*):ti,ab,kw	50945
#15	(clip*):ti,ab,kw	4038
#16	(hemoclip*):ti,ab,kw	142
#17	(endoclip*):ti,ab,kw	82
#18	(dental floss):ti,ab,kw	966
#19	(forcep*):ti,ab,kw	2101
#20	(grasping forcep*):ti,ab,kw	106
#21	(thread*):ti,ab,kw	1401
#22	(string*):ti,ab,kw	2820
#23	(snare*):ti,ab,kw	632
#24	(preloop*):ti,ab,kw	10
#25	(double scope*):ti,ab,kw	598
#26	(transnasal endoscope*):ti,ab,kw	100
#27	(magnetic*):ti,ab,kw	51135

#28	(anchor*):ti,ab,kw	3767
#29	(sinker*):ti,ab,kw	5
#30	(band*):ti,ab,kw	15949
#31	(ring thread*):ti,ab,kw	46
#32	#9 or #10 or #11 or #12 or #13 or #14 or #15 or #16 or #17 or #18 or #19 or #20 or #21 or #22 or #23 or #24 or #25 or #26 or #27 or #28 or #29 or #30 or #31	293154
#33	(random*):ti,ab,kw	1401841
#34	MeSH descriptor: [Randomized Controlled Trial] explode all trees	34
#35	#33 or #34	1401841
#36	#8 and #32 and #35	990

Table S4. Sensitivity analysis of procedure time data

Study Omitted	Meta-analysis
Bi et al (2024)	SMD: -0.14, 95% CI: -0.29 to 0.01
Kinoshita et al (2024)	SMD: -0.24, 95% CI: -0.49 to 0.01
Hasatani et al (2022)	SMD: -0.22, 95% CI: -0.49 to 0.05
Nagata et al (2021)	SMD: -0.19, 95% CI: -0.41 to 0.03
Yoshida et al (2018)	SMD: -0.27, 95% CI: -0.54 to -0.01
Ban et al (2018)	SMD: -0.27, 95% CI: -0.51 to -0.04
Ahn et al (2013)	SMD: -0.27, 95% CI: -0.49 to -0.05

Abbreviations: SMD: standardized mean difference, CI: confidence interval.

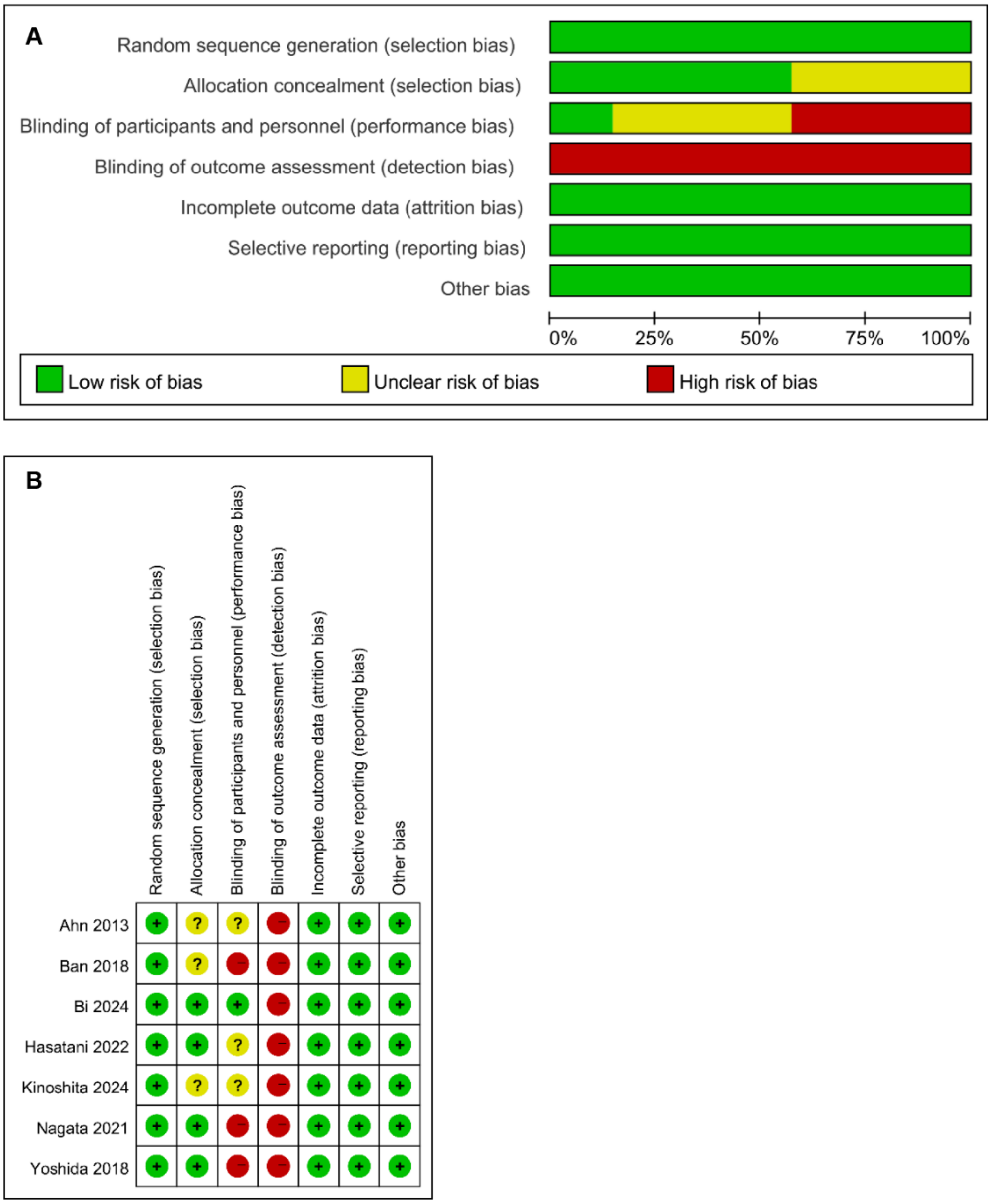

Fig. S1 Comprehensive summary of the methodological quality of the included RCTs

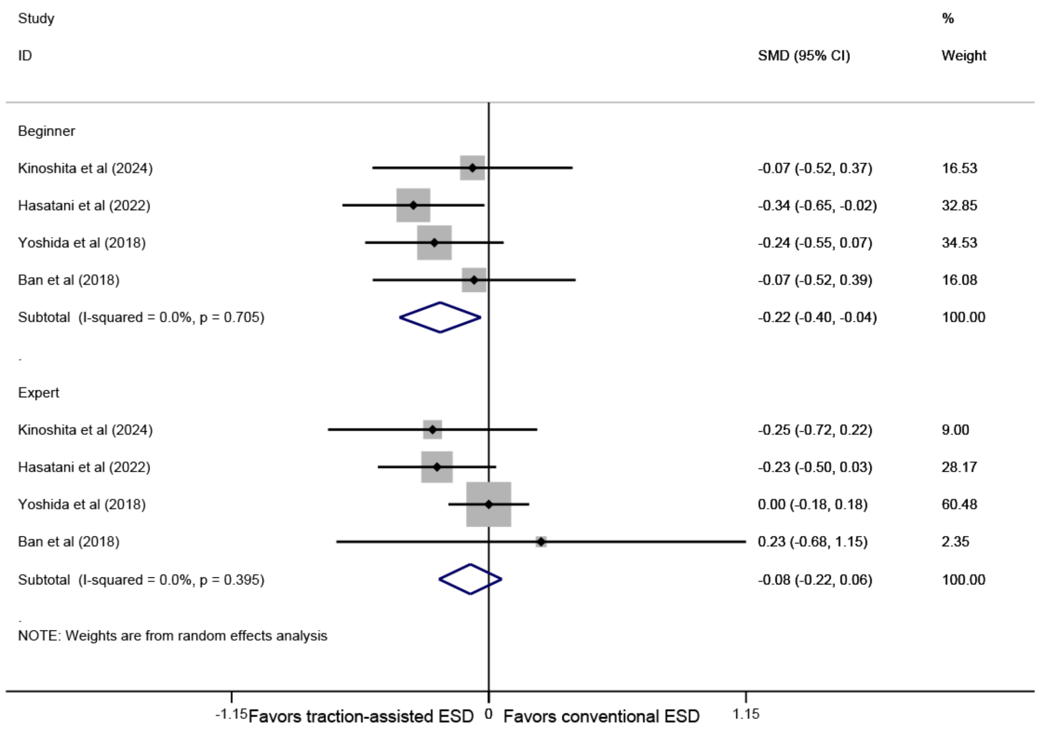

Fig. S2 Subgroup analysis of procedure time stratified by operator experience (beginner vs. expert)

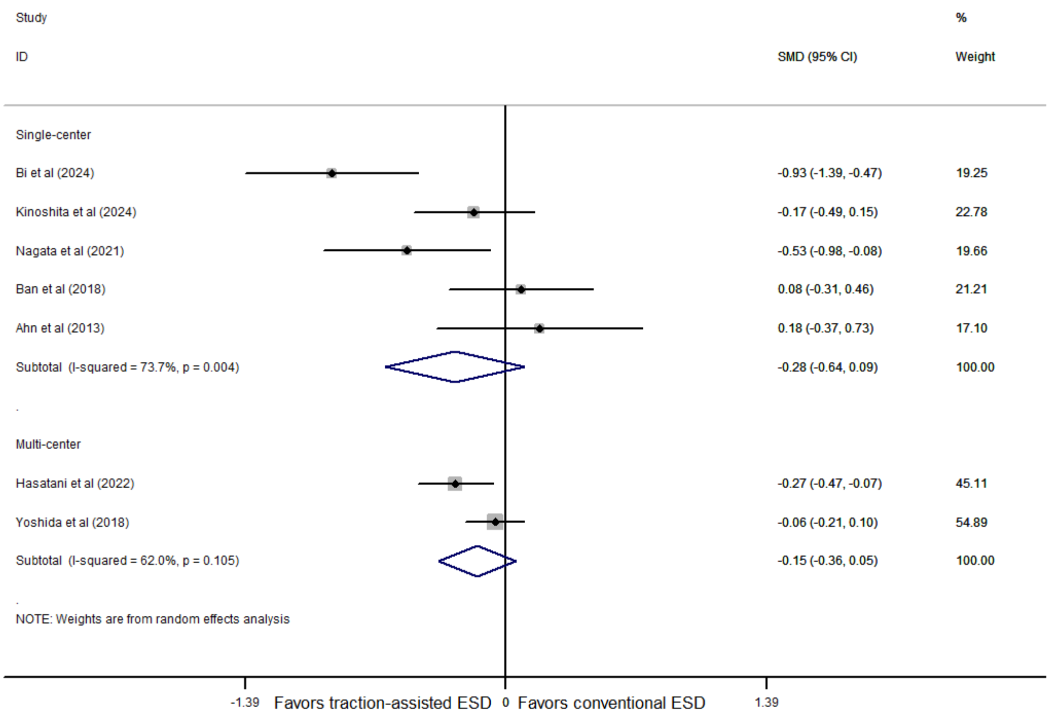

Fig. S3 Subgroup analysis of procedure time stratified by study design (single- vs. multi-center)

Study or Subgroup	Traction-assisted ESD Events	Traction-assisted ESD Total	Conventional ESD Events	Conventional ESD Total	Weight	Risk Ratio M-H, Random, 95% CI
Ahn 2013	26	26	25	25	0.5%	1.00 [0.93, 1.08]
Ban 2018	49	49	55	55	1.8%	1.00 [0.96, 1.04]
Bi 2024	40	40	40	40	1.1%	1.00 [0.95, 1.05]
Hasatani 2022	186	186	192	192	24.0%	1.00 [0.99, 1.01]
Kinoshita 2024	73	73	75	75	3.7%	1.00 [0.97, 1.03]
Nagata 2021	40	40	40	40	1.1%	1.00 [0.95, 1.05]
Yoshida 2018	319	319	316	316	67.7%	1.00 [0.99, 1.01]
**Total (95% CI)**		**733**		**743**	**100.0%**	**1.00 [0.99, 1.01]**
Total events	733		743			

Heterogeneity: Tau² = 0.00; Chi² = 0.00, df = 6 (P = 1.00); I² = 0%

Test for overall effect: Z = 0.00 (P = 1.00)

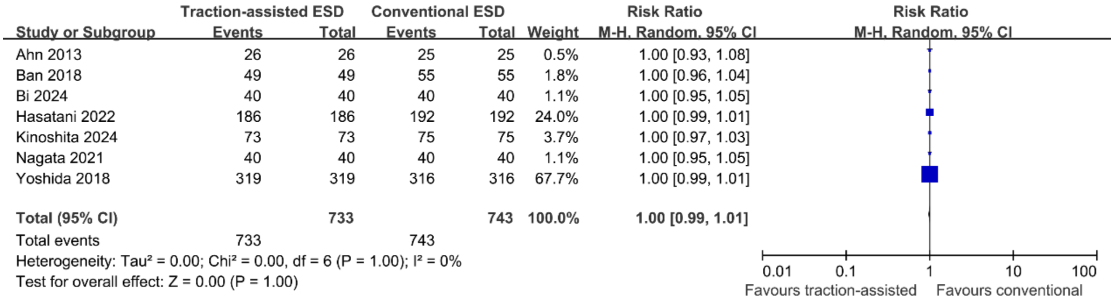

Fig. S4 Forest plot of *en bloc* resection rates between traction-assisted and conventional ESD
